# The Production, Assessment, and Utility of 3D-Printed Video Laryngoscopes in Eastern India: A Low-Cost Alternative to Conventional Video Laryngoscopes

**DOI:** 10.7759/cureus.60386

**Published:** 2024-05-15

**Authors:** Aditya Lal Vallath, Suhasini Krishnan, Ena Skikic, Tania Das, Snigdha Banerjee, Aryapriyo Chatterjee, Indraneel Dasgupta

**Affiliations:** 1 Emergency Medicine, Peerless Hospital and B.K. Roy Research Center, Kolkata, IND; 2 Internal Medicine, Dubai Academic Health Corporation, Dubai, ARE; 3 Trauma and Orthopaedics, Peerless Hospital and B.K. Roy Research Center, Kolkata, IND; 4 Clinical Pharmacology, Peerless Hospital and B.K. Roy Research Center, Kolkata, IND

**Keywords:** video-laryngoscope, emergency medicine, teaching in emergency medicine, low cost healthcare, ‎3d printing, rapid sequence intubation (rsi), direct laryngoscope, simulation in medical education, airway intubation, 3d printed video laryngoscope

## Abstract

Introduction

Recognizing the limitations of traditional direct laryngoscopes, particularly in difficult airway situations, video laryngoscopy has emerged as a potentially safer and more effective alternative. This study evaluated the utility of two 3D-printed video laryngoscopes: a standard geometry video laryngoscope (SGVL), resembling the traditional Macintosh blade, and a hyper-angulated video laryngoscope (HAVL) with a more curved design. Their performance was compared to a standard Macintosh direct laryngoscope across various intubation parameters. By leveraging the cost-effectiveness of 3D printing with polylactic acid, the study aimed to assess the potential of this technology to improve airway management across diverse clinical settings and varying levels of physician expertise.

Methods

This prospective randomized crossover study compared the effectiveness of 3D-printed video laryngoscopes (VL) and a standard direct laryngoscope in intubation. After obtaining IRB approval, physicians from various specialties across multiple centers participated. Participants received training on SGVL, HAVL, and DL intubation using an instructional video and hands-on practice. The training was standardized for all participants. The primary outcome measures were time to successful intubation, number of attempts, and time to visualize vocal cords. Participants were randomized to use all three laryngoscopes on a manikin, with a maximum of two attempts per scope. A 30-minute break separated each laryngoscope evaluation. Successful intubation was defined as the single insertion of each laryngoscope and bougie, followed by endotracheal tube placement and confirmation of lung inflation.

Results

Ninety-eight doctors, mostly from the EM team (73.5%) and ICU team (23.4%). Teams consist of consultants, residents, and medical officers of the concerned departments. Forty-eight of the participants (49%) were novice operators (<25 intubations). Successful first-attempt intubation in those with <1 year of experience with intubation (n=33) was highest for SGVL (97%) compared to DL (82%) and HAVL (67%). Participants who learned intubation through self-directed learning exhibited a higher acceptance of VL and achieved 100% success on their first attempt. Among those who followed modules or workshops, 97% had successful first-attempt intubation with VL. The average time taken to visualize the vocal cords was lower in SGVL compared to DL (5.6 vs. 7.5 seconds) (p<0.001). The HAVL also had a lower average time compared to the DL (7.1 vs. 7.5 secs) (p<0.001). However, the time taken to intubate using DL (24.2 ±8.7 sec) was similar to SGVL (28.1 ±13 sec). Lastly, the intubation time using HAVL was the longest (49.6 ±35.5 sec). The time to intubate with DL and SGVL had Spearman's rho of 0.64 (p<0.001), and DL and HAVL had 0.59 (p<0.001).

Conclusions

The ease of use and its cost-effective nature make 3D-printed VLs beneficial in situations where traditional VLs may not be available, especially in simulation and training.

## Introduction

The use of video laryngoscopes in an emergency department (ED) setting can ease intubation by providing improved visualization of the glottis, thus decreasing the risk of complications [1]. However, video laryngoscopes (VL) are not available in resource-limited settings. Fused deposition modeling (FDM) is one of the most cost-effective methods of 3D printing, wherein polymer filaments are melted and extruded through the nozzle of the 3D printer on a build plate as per the coded instructions [2]. The COVID-19 pandemic caused a global shortage in biomedical supplies, specifically personal protective equipment, ventilators, and oxygen valves. To meet this growing demand, mass 3D printing of these devices using polymer filaments such as polylactic acid (PLA) proved to be a highly convenient and viable option [3]. It is biocompatible and capable of undergoing repeated sterilization without risk of alterations in its physical and chemical structure, maintaining its functionality [4]. The 3D-printed medical devices can be sterilized chemically and using UV light. This reusability is valuable in resource-constrained settings.

In this study, we produced and assessed the utility of two styles of 3D-printed video laryngoscopes, standard and hyperangulated, against a conventional direct laryngoscope across residents and specialists of various skills in intubation and airway management. This study aims to evaluate the potential benefits of 3D-printed video laryngoscopes compared to traditional direct laryngoscopes during simulated intubation on a manikin. We will measure the time taken to successfully intubate, the number of attempts required, and the time needed to visualize the vocal cords. Due to the nonavailability of video laryngoscopes at the study site as well as at the sites participants were recruited from, the 3D-printed devices were compared against direct laryngoscopes.

## Materials and methods

**Figure 1:**
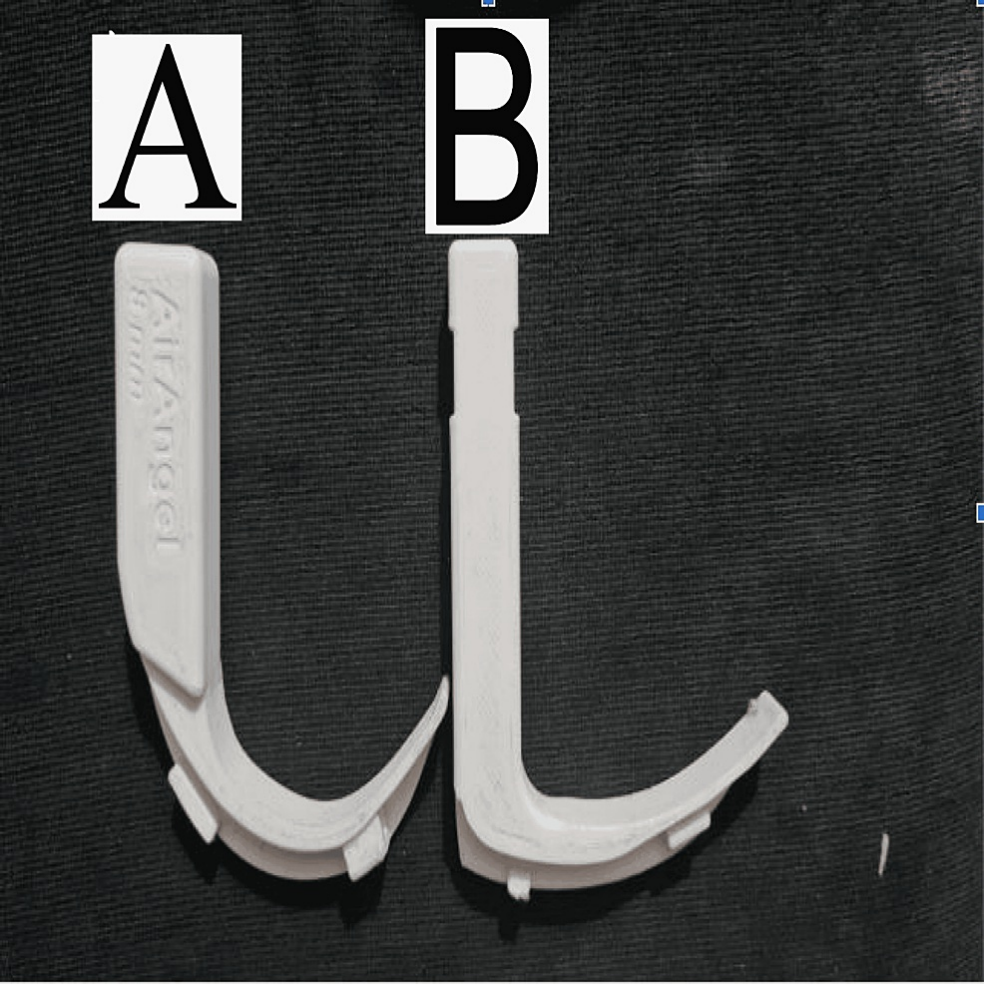
3D printed air angel standard adult hyperangulated blade (A) and standard geometry video laryngoscope (B)

**Figure 2:**
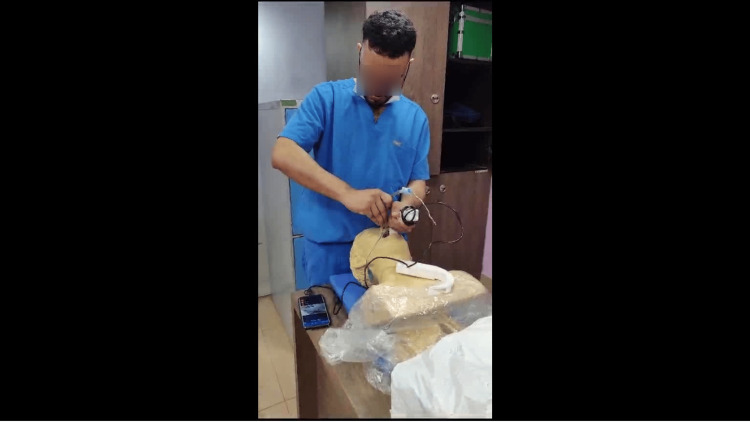
3D-printed standard video laryngoscope in use. The video output is from a Samsung s20+ Android phone connected via a Type C data port

After obtaining successful IRB approval (IRB name: Peerless Hospitex Hospital and Research Center Limited Clinical Research Ethics Committee, Approval Number: PHH&RCL-CREC/S01/2023), a prospective observational study was conducted with participants from multiple specialties across various centers. These centers lacked access to commercial VLs and were identified based on the inclusion criteria. The inclusion criteria included consenting physicians from emergency medicine, ICU, internal medicine, and surgery across multiple centers. Individuals unable to provide consent or untrained staff were excluded. The 3D print file and characteristics can be found in Table 6 in the Appendix. To assess the correlation between the video laryngoscope and hyperangulated video laryngoscope with the direct laryngoscope, an alpha level of 0.05, a beta level of 0.2, and a correlation coefficient of 0.25 was used using the formula N = [(Zα+Zβ)/C]2 + 3. The total number of participants calculated for this study was 98. The primary objectives of this study were to compare and correlate the following parameters among the three scopes: the time taken to visualize vocal cords, the time taken to intubate, and the number of attempts. Figure 1 shows 3D printed air angel standard adult hyperangulated blade. Figure 2 shows 3D-printed standard video laryngoscope in use.

An 8mm fiber optic waterproof videoscope (2 m) with a USB type C connector was used (manufactured by Wukuma Inc.). The videoscope was connected to a Samsung S20+ Android phone, which provided the video output. The characteristics of the videoscope are listed in Table 7 in the Appendix.

The 3D-printed video laryngoscope used was made of polylactic acid (PLA) plastic for its biocompatible properties. All voluntary participants were included in the study. As a part of the study procedure, participants were briefed on the study objective, consent was obtained, and training was provided using an instructional training video as well as a hands-on demo using the standard VL, hyperangulated VL, and a gum elastic bougie for intubation. After the training, participants used all scopes twice on the manikin in an untimed run on an electronic airway intubation model. After completing the training module, the participants were asked to complete a questionnaire to evaluate their confidence and skills in various airway management procedures, as well as indicate their learning sources in Table 8 in the Appendix. The questions asked are listed in the Appendix. During the final evaluation, the primary outcomes listed in Table 9 in the Appendix were recorded. The primary outcome was assessed by an independent observer blinded to group assignment. The participants were randomized to three different stations. Each of the three stations had either a DL, standard VL, or hyperangulated VL. The participants were asked to complete the intubation task and were shifted to the next station after successful intubation or if they failed more than two attempts. Before starting with the next station, participants were told to wait for 30 minutes before starting to mitigate carryover effects. ﻿

Successful intubation was considered the single insertion of a laryngoscope blade into the mouth, along with the single insertion of a bougie into the trachea, followed by the placement of an endotracheal tube into the manikin trachea with the cuff placed distally to the vocal cords. Successful intubation was indicated by the flashing of the success light as well as the inflation of the manikin lungs on positive pressure ventilation by a bag valve mask. Any deviation from the previously mentioned conditions is considered an attempt. Each operator was allowed two attempts. Failure to intubate successfully after two attempts was considered a failed intubation and the end of the test. The evaluation assessed participants using all three laryngoscopes in bougie-assisted intubation. If two attempts or less were taken, then the average time was calculated and recorded.

Statistical methods

Descriptive statistical tests were used to calculate the results. The Shapiro-Wilk test was performed to determine the distribution of the data. Given the small sample size, Spearman's rho was used to assess correlations between intubation times with different devices; a p-value of <0.05 was considered to be significant.

## Results

Table 1 shows the number of intubations performed.

**Table 1:** Number of intubations performed The total number of participants included in this study was 98; 49% had performed 25 intubations or less throughout their careers. The average confidence level (scaled from 0 to 10) before the training session for direct laryngoscopy (DL) was 7.2, whereas for video laryngoscopy (VL), it was 4.4.

Number of Intubations Performed by Participants in Their Career Till Date	No of Participants	Total %
0-25	48	49.0%
25-50	27	27.6%
75-100	23	23.5%

As demonstrated in Table 2, individuals who learned intubation through self-direction or using journals comprised 64.3% of the participants. Of those, all participants (100%) had a successful first attempt at intubating using Standard Geometry VL. Moreover, the success rates for first attempts using DL and hyperangulated VL were 80.9% and 63.5%, respectively. Likewise, participants who utilized modules, workshops, or lectures were 71.4%. Among them, 97.1%, 82.9%, and 62.9% had a successful first attempt with intubation using VL, DL, and hyperangulated VL, respectively. We can conclude that Standard Geometry VL is the easiest to learn among the three options and yields satisfactory first-pass success rates regardless of the participants’ preferred learning method. Table 3 shows the success rates of participants based on their preferred method of learning.

**Table 2:** Success rates of participants based on their years of experience in airway management The successful first-attempt intubation rates were higher in Standard Geometry VL 32/33 (96.96%) among individuals with less than one year experience in airway management compared to DL 27/33 (81.81%) and hyperangulated video laryngoscopy (HAVL) 22/33 (66.66%). Similarly, individuals with more than three years of experience had higher success rates with Standard Geometry VL 28/28 (100%) compared to DL 24/28 (85.71%) and Hyperangulated VL 17/28 (60.71%).

		Years of Experience in airway management			Total (n=98)	Number of successful intubation (%)
	No of attempts taken during intubation	Less than 1 year (n=33)	1-3 years (n=37)	More than 3 years (n=28)		
Direct Laryngoscope	1	27 (33.7%)	29 (36.2%)	24 (30%)	80	98 (100%)
	2	6 (33.3%)	8 (44.4%)	4 (22.2%)	18	
Standard Geometry Video Laryngoscope	1	32 (33.3%)	36 (37.5%)	28 (29.1%)	96	98 (100%)
	2	1 (50%)	1 (50%)	0	2	
Hyperangulated Video Laryngoscope	1	22 (36%)	22 (36%)	17 (27.8%)	61	85 (86.7%)
	2	6 (25%)	9 (37.5%)	9 (37.5%)	24	
	3	0 (0%)	1 (100%)	0 (0%)	1	
	4	3 (37.5%)	4 (50%)	1 (12.5%)	8	
	5	2 (50%)	1 (25%)	1 (25%)	4	

**Table 3:** Success rates of participants based on their preferred method of learning

		Self-Directed/Journal		Module/Workshop/Lecture		Total (n=98)
	No of attempts	No (n=35)	Yes (n=63)	No (n=28)	Yes (n=70)	
Direct Laryngoscope	1	29 (82.8%)	51 (80.9%)	22 (78.5%)	58 (82.8%)	80 (81.6%)
	2	6 (17.1%)	12 (19%)	6 (21.4%)	12 (17.1%)	18 (18.3%)
Standard Geometry Video Laryngoscope	1	33 (94.2%)	63 (100%)	28 (100%)	68 (97.1%)	96 (97.9%)
	2	2 (5%)	0	0	2 (2.8%)	2 (2%)
Hyperangulated Video Laryngoscope	1	21 (60%)	40 (63.4%)	17 (60.7%)	44 (62.8%)	61 (62.2%)
	2	11 (31.4%)	13 (20.6%)	7 (25%)	17 (24.2%)	24 (24.4%)
	3	0 (0%)	1 (1.5%)	1 (3.5%)	0 (0%)	1 (1%)
	4	2 (5.7%)	6 (9.5%)	2 (7.1%)	6 (8.5%)	8 (8.1%)
	5	1 (2.8%)	3 (4.7%)	1 (3.5%)	3 (4.2%)	4 (4%)

The average time taken to visualize the vocal cords was significantly lower with Standard Geometry VL compared to DL (5.6±2.7 vs. 7.4±3.2 seconds) (p<0.001). The hyperangulated VL also had a significantly lower average time compared to the DL (7.1±3.9 vs. 7.5±3.3 seconds) (p<0.001). Furthermore, the average time taken to perform intubation using DL (24.2±8.7 seconds) was similar to the average time taken to perform intubation using Standard Geometry VL (28.1±13 seconds) (p<0.001). Lastly, the average time taken to perform intubation using hyperangulated VL was the longest among the three techniques (49.6±35.5 seconds) (p<0.001). Table 4 shows the average time taken to visualize vocal cords and intubate.

**Table 4:** Average time taken to visualize vocal cords and intubate Shapiro-Wilk test was performed, and it was found to be normally distributed.

	Total (n=98)	Mean (seconds)	Std. Deviation (seconds)
Time taken to visualize vocal cords			
Direct Laryngoscope	98 (100%)	7.5	3.3
Standard Geometry Video Laryngoscope	98 (100%)	5.6	2.7
Hyperangulated Video Laryngoscope	95 (96.9%)	7.1	3.9
Time taken to intubate			
Direct Laryngoscope	98 (100%)	24.2	8.7
Standard Geometry Video Laryngoscope	98 (100%)	28.1	13.0
Hyperangulated Video Laryngoscope	92 (93.8%)	49.6	35.5

Table 5 shows that the time taken to visualize VC using direct laryngoscopy (DL) and the time taken to visualize VC using Standard Geometry VL have a correlation coefficient of 0.637 (p=0.000) p<0.001. Similarly, the correlation between the time taken to visualize VC with DL and the time taken to visualize VC with hyperangulated VL is 0.593, (p=0.000) p<0.001. The table also demonstrates the correlation between the time taken to perform intubation using DL and the time taken to intubate using Standard Geometry VL, which has a Spearman's rho of 0.541 (p=0.000) p<0.001. Additionally, it shows the correlation between the time taken to intubate with DL and the time taken to intubate with hyperangulated VL, which is 0.459, p<0.001. The results of the Spearman correlation analysis for the three techniques are presented in Table 5.

**Table 5:** Results of correlation analysis Spearman's rho was used to assess correlations between intubation times with different devices, a p-value of <0.05 was significant.

Time taken to visualize vocal cords		Standard geometry video laryngoscope	Hyperangulated video laryngoscope
Direct Laryngoscope	Correlation Coefficient	0.637	0.593
	p-value	<0.001	<0.001
Time taken to intubate		Standard geometry video laryngoscope	Hyperangulated video laryngoscope
Direct Laryngoscope	Correlation Coefficient	0.541	0.459
	p-value	<0.001	<0.001

All participants reported a satisfaction rate of 100% after the training, and 78.6% indicated that they preferred to use 3D-printed video laryngoscopes in the future.

## Discussion

Our study explores the performance of 3D-printed hyperangulated and standard geometry video laryngoscopes compared to direct laryngoscopes to optimize airway management by physicians of varied experience levels. Physicians with less than one year of experience in airway management successfully intubated in their first attempt with standard geometry VL more frequently than with DL or hyperangulated VL (96.96% vs. 81.81% vs. 66.66%, respectively). Similar trends were noted in individuals with more than three years of experience in airway management, with the highest number of successful intubations performed with standard geometry VL (100%), followed by DL (85.71%), and hyperangulated VL (60.71%). Compared to DL, VLs offer easier visualization of the vocal cords and relevant anatomy due to the camera and screen, improving success rates. Additionally, the screen allows for remote guidance by senior physicians during challenging intubations, potentially improving safety and outcomes. The use of a bougie further simplifies the process of inserting the endotracheal tube, contributing to improved intubation performance, especially when used in conjunction with video laryngoscopy. [5]. The device setup allows for an easy up-close view of the glottis, bypassing the need for elaborate positioning of the oral, laryngeal, and pharyngeal axes. Notably, the type of blade present may influence success, as channeled VLs have proven superior in ease of use and intubation safety [6]. 

Numerous studies have been conducted to compare the efficacy of VL with DL in various in-hospital settings. VL has outperformed DL, according to studies conducted across general wards, intensive care units, and emergency departments, in terms of first-pass success [5-10] and fewer intubation attempts [9]. The majority of the participants in the study were juniors in their respective fields and had less intubation expertise. Our results revealed that standard geometry VL had 100% success in self-directed learners and 97.1% success in module/workshop/lecture-based learners. Prekker et al. and other similar studies found that VL increased first-pass intubation when attempted by physicians of all levels of experience, with its greatest benefit reaped by the least skilled participants [5,7,8,10]. A retrospective study of intubations performed by emergency medicine residents advancing through their training program showed that the learning curve and skill advancement were more pronounced when using video laryngoscopy [10]. However, evidence is limited, and further RCTs assessing the learning curve of performing video laryngoscopy are needed. On the other hand, some studies have reported no significant advantage of using VL when considering intubation success rates, time to intubation, and survival rates. This may be largely due to operators’ lack of prior VL experience, inadequate use of muscle relaxants and anesthetic drugs, and, mainly, the difficulty of intubating with an indirect view of the glottis without adequate anatomical alignment. They noted a lack of standardized patient selection or measures of intubation expertise levels across studies and acknowledged the possibility of physicians selectively utilizing VL for difficult airways and DL for less difficult ones [11,12]. 

Nalubola et al. demonstrated that the time taken to intubate was longer with DL than VL, with a mean difference (DL-VL) of 14.58 seconds (95% CI = 5.61, 23.54) [6]. In our study, the time taken to intubate DL (mean of 24.18±8.72 seconds, p<0.001) was similar to Standard Geometry VL (mean of 28.13±13 seconds, p<0.001). We hypothesize that the time to intubate is shorter in our study as we provided learning materials, a brief trial period was given for the participants to familiarize themselves, and the study was conducted in a Manikin model, which eliminated external factors. Hyperangulated VL had a longer intubation time (mean value of 49.58±35.45 seconds, p<0.001). This is due to the need to perform more complex maneuvers while the anatomical structures are unaligned.

Currently, the role of 3D printing in the medical field spans a range of individual projects and trials [13,14]. 3D printing technology is used to mitigate challenges worldwide, such as rapidly and cheaply producing respirators, face shields, nasopharyngeal swabs, ventilator adapters, mask adapters, emergency quarantine facilities, training models, etc. [15]. Notably, 3D-printed video laryngoscopes were used during the COVID-19 pandemic and for military purposes in austere environments [16]. VL was particularly useful during the pandemic as it allowed for a distance to be held between the incubator and the COVID-positive patient, decreasing the risk of infection for frontline workers [5]. The benefits of using 3D-printed VLs include cost-effectiveness and ease of production, allowing for a quicker supply of these critical airway management tools to communities in need. Presently, we lack data, or the usage of VL is limited to areas with high medical resources [17]. The 3D-printed VLs can increase accessibility, benefiting areas with scarce medical resources and economic disadvantages. In addition to disaster relief, we believe that 3D-printed VLs could play a major role in airway management training for medical students and residents. By using biocompatible PLA with proper sourcing and manufacturing, the problems of structural collapse or fragmentation can be avoided. This biopolymer is inexpensive to produce and has extensive applications in modern medicine across numerous fields, which include orthopedics, dentistry, infectious diseases, oncology, and cardiology [4].

The strengths of our study lie in the guided session, which included an instructional video along with a manikin practice session held on the same day as the study. The training session was targeted at novice operators and the particular challenges they face in using the scopes. The results reported were representative of current healthcare professionals. Additionally, there was no difficulty disclosed by the participants concerning learning and adapting to using video laryngoscopy. On the other hand, limitations to this study exist mainly in the limited sample size of the participants in general and participants without prior DL experience. More experienced physicians were lacking in this study. The research also lacks variations in anatomy that could occur in the population and real-world scenarios, namely complications, equipment failure, or camera lens obstruction.

## Conclusions

This study explored the use of 3D-printed video laryngoscopes (VLs) in airway management by physicians of varied specialties and expertise. Video laryngoscopy shows potential in novice operators. Furthermore, we believe medical education and training programs would highly benefit from the introduction of 3D-printed components in simulation labs and workshops. With rapid advances in polymer sciences and 3D printing, further iterations of the video laryngoscope can be tailored to its specific requirements and use. Further research is needed to evaluate the safety and efficacy of 3D-printed video laryngoscopes in clinical practice. However, the potential benefits of this technology are significant, and it is likely to play an increasingly important role in airway management in the future.

## Appendices

**Table 6:** 3D print characteristics

Name of Blade - Air Angel Standard Adult Hyperangulated Blade							
Creator	Bryan Archpru and Steven Bazan						
Print Material	PLA+ (Poly Lactic Acid)						
Print characteristics	Nozzle diameter 0.4mm	Layer thickness: 0.2mm	Number of perimeters: 4	Infill: 30%	Bed Temperature: 50°C	Type of printing process: FDM	
							Website reference	https://www.airangelblade.org/							
Name of Blade- Standard Geometry Video Laryngoscope								
Creator	Thomas Große							
Print Material	PLA+ (Poly Lactic Acid)							
Website reference	https://www.thingiverse.com/thing:3967800							
Print characteristics	Nozzle Diameter: 0.2mm	Layer thickness: 0.2mm	Number of perimeters: 4	Infill: 100%	Bed Temperature: 50 °C	Type of printing process: FDM		

**Table 7:** Videoscope features

Lens Diameter	8.0mm
Resolution	1280x720
Sensor size	1/9 inch
Frame rate	30 FPS
View Angle	67 Degrees
Water Proof Level	IP67
Operating Temperature	0-70 Deg C
Cable Length	2 mts
Power Supply	5V DC via USB C
Operating System	Android

**Table 8:** Airway evaluation self-questionnaire

S no.	Questions Scored from 1-10	
1	How confident are you in DL?	
2	How confident are you in Emergency Surgical Airway (Cricothyroidotomy)?	
3	How confident are you in Awake intubation?	
4	How confident are you in Use of a fiberoptic bronchoscope (asleep)?	
5	How confident are you in Videolaryngoscopy?	
6	How confident are you in Supraglottic airway use?	
7	How confident are you in Intubation through a supraglottic airway device?	
8	How confident are you in Self-inflating bag/ mask ventilation?	
9	How confident are you in Rapid Sequence Induction?	
10	How confident are you in Retrograde Intubation?	
11	How confident are you in Extubation of the difficult airway?	
12	What is the main form of teaching at your institute with regards to airway management?	Lecture series
		Compulsory workshop
		Optional workshop
		Designated airway module during training
		Self-taught
		Self-directed learning outside the OR (e.g., taking an e-learning module or watching a DVD, reading a journal, unsupervised
		Supervised teaching

**Table 9:** Assessment questionnaire

Did you receive the specified training before this assessment (DL with Bougie and VL with Bougie)? Yes/No
Are you satisfied with the training provided? Yes/No
Time taken to visualize VC with DL
Time taken to visualize VC with Hyperangulated VL
Time taken to visualize VC with Standard Geometry VL
Time taken to intubate with DL
Time taken to intubate with Hyperangulated VL
Time taken to intubate with Standard Geometry VL
Number of attempts (DL)
Number of attempts (Standard Geometry VL)
Number of attempts (Hyperangulated VL)
Was intubation successful (DL)?
Was intubation successful (Standard Geometry VL)?
Was intubation successful (Hyperangulated VL)?
After this training, would you consider using a 3D-printed VL for first-attempt intubation or difficult intubation? I will use VL for both standard and difficult intubation. I will only use VL for difficult intubation. I will only use DL for all intubations.
